# The regulatory role of prostaglandin E_2 _in liver (patho) physiology is controlled at its site of synthesis and its action on the receptors

**DOI:** 10.1186/1476-5926-2-S1-S35

**Published:** 2004-01-14

**Authors:** Peter Dieter, Roland Scheibe, Yefgeniya Bezugla, Egbert Matthé, Sandra Schuch, Lars Treffkorn, Brigitte Bernard, Sabine Kamionka, Angelika Kolada

**Affiliations:** 1 Institute of Physiological Chemistry, Medical Faculty Carl Gustav Carus, TU Dresden, Fetscherstrasse 74, D-01307 Dresden, Germany

## Introduction

Among the hormone class of the eicosanoids, PGE_2 _plays a predominant role in liver (patho) physiology. Liver-specific responses, like regulation of blood glucose homeostasis, sinusoidal blood flow within the liver, properties of the transendothelial barrier within the liver, synthesis and release of important other mediators like cytokines, growth factors or nitric oxide, and liver fibrogenesis have been shown to be mediated or regulated by PGE_2 _[1]. Within the liver, the main producers of PGE_2 _are the Kupffer cells. The synthesis of PGE_2 _in Kupffer cells is controlled at multiple levels. The action of PGE_2 _on its target cells is mediated by 4 classes of PGE_2 _receptors (EP1, EP2, EP3, EP4). Each of these receptors converts the information of PGE_2 _by different intracellular signal pathways to a specific cellular response [2].

## Methods

Liver nonparenchymal cells (endothelial cells, Kupffer cells, stellate cells) are isolated from male rat livers by a pronase/collagenase perfusion. Experiments are performed with cells kept in primary cultures [1].

## Results and Discussion

Isolated liver nonparenchymal cells (endothelial cells, Kupffer cells, stellate cells) are characterized by different markers (Table 1).

**Table 1:** Characterization of endothelial cells (EC), Kupffer cells (KC) and stellate cells (SC) by different markers.

Marker	ED-1	Latex Beads	Ac-LDL	vWF	Reca -1	CD 31	SMA	Desmin
EC	neg	neg + pos	neg + pos	pos	pos	pos	neg	neg
KC	pos	pos	pos	neg	neg	neg	neg	neg
SC	neg	neg	neg	neg	neg	neg	pos	pos

**Figure 1:**
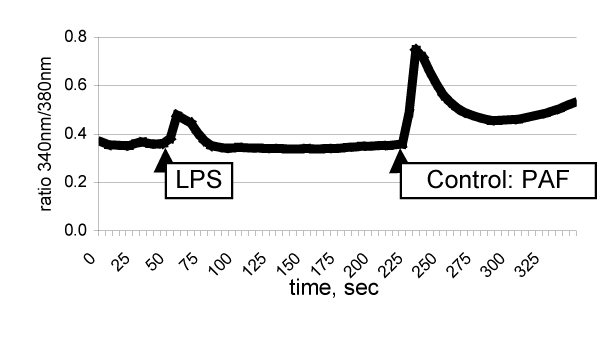
Intracellular free calcium after LPS and platelet activating factor (PAF).

**Figure 2:**
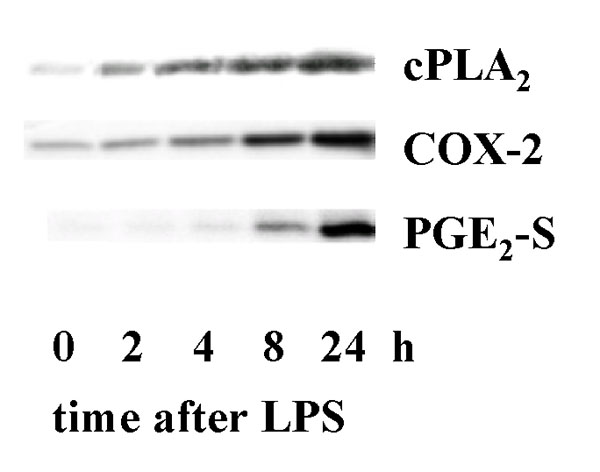
LPS-induced expression of cPLA_2_, COX-2 and PGE_2_-synthase (S).

The fast synthesis of PGE_2 _in Kupffer cells (induced by, e.g., platelet activating factor, zymosan, calcium ionophore) requires a sustained increase of cellular calcium (Fig. 1). The delayed synthesis of PGE_2 _in Kupffer cells (induced by, e.g., LPS) is paralleled by a transient increase of cellular calcium (Fig. 1), and requires a *de novo */ enhanced expression of cytosolic phospholipase A_2 _(cPLA_2_), cyclooxygenase (COX)- 2 and PGE_2 _synthase (Fig. 2).

**Figure 3:**
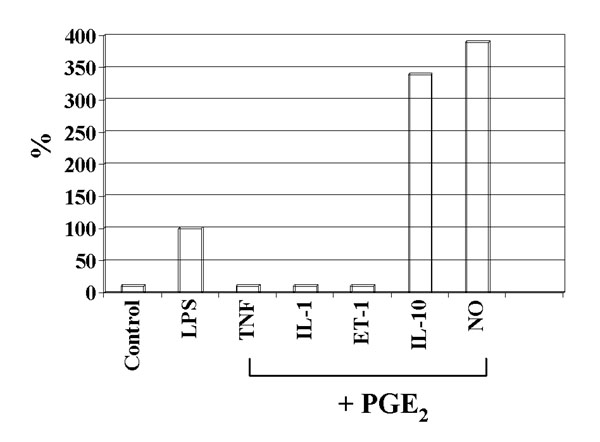
Effect of PGE_2 _on LPS-induced formation of TNF-alpha, IL-1, ET-1, IL-10 and NO.

**Figure 4:**
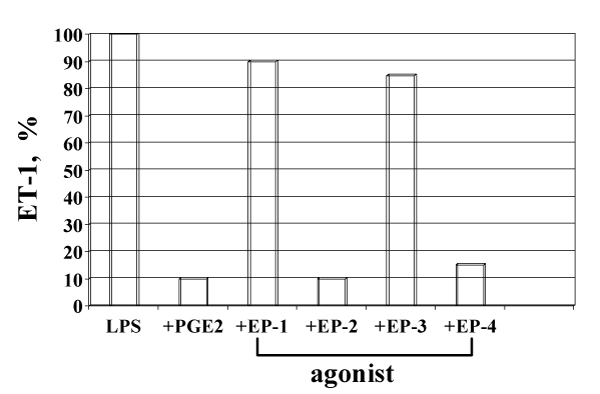
Effect of PGE_2_-receptor agonists (EP -1/-2/-3/-4) on LPS-induced release of ET-1.

Besides eicosanoids, LPS induces in Kupffer cells the release of other mediators, including IL-1, IL-10, TNF-alpha, ET-1, and NO (1). The release of IL-1, TNF-alpha and ET-1 is totally suppressed by PGE_2_, the release of IL-10 and NO (1) is enhanced by PGE_2 _(Fig. 3). The regulation of the synthesis of IL-1, IL-10, TNF-alpha (Fig. 4) and ET-1 in Kupffer cells by PGE_2 _is mediated by EP-2 and EP-4, as demonstrated by the use of PGE_2_-receptor-specific agonists (EP-1/-2/-3/-4:ONO-DI-004/-AE1-259/-AE-248/-AE1-329).

**Figure 5:**
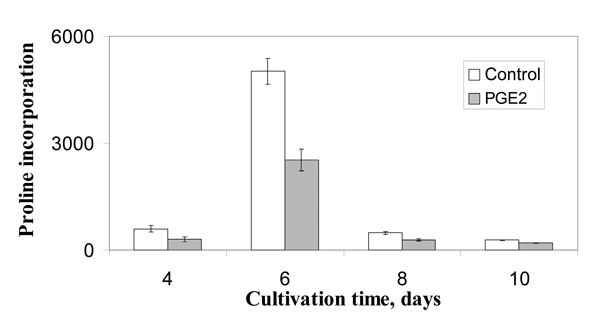
Effect of PGE_2 _on collagen synthesis (proline incorporation) in stellate cells.

PGE_2 _inhibits proliferation, transdifferentiation and collagen synthesis (Fig. 5) of Stellate cells.

## Conclusions

**Figure 6:**
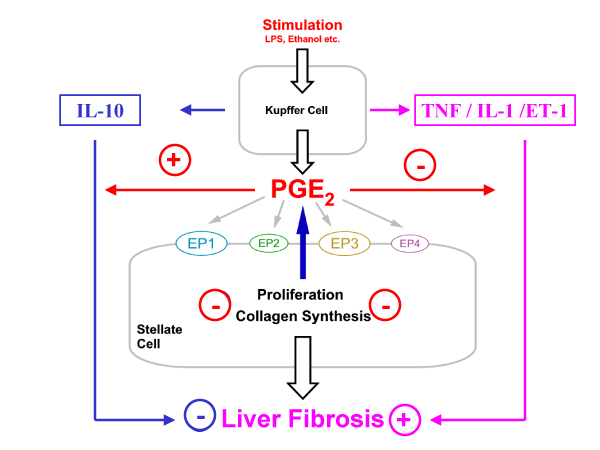
PGE_2_: A potent physiological suppressor of liver fibrosis.

PGE_2_, produced by Kupffer cells, is a potent physiological suppressor of liver fibrosis (Fig. 6).
